# The Book World of Medicine and Science

**Published:** 1905-02-18

**Authors:** 


### The Book World of Medicine and Science.

A Treatise on Bright's Disease and Diabetes. By James Tyson, M.D. Second edition, including a section on the Ocular Changes in Bright's Disease and in Diabetes, by G. E. de Schweinitz, M.D. (London: Rebman, Limited. 1904. Pp. 381; 43 illustrations; 7 coloured Plates. Price 17s. 6d. net.)

A second edition of this book has been published after over 20 years, during a considerable portion of which time it has been out of print. The author has nevertheless spared no pains to modernise the work, and has produced a text-book which is both reliable and complete, containing as it does the opinions of every important writer on the subject. While, perhaps, the recapitulation of the diverse classifications of the conditions included under Bright's Disease by different authors might have been considerably curtailed, few would be disposed to abridge the writer's lucid if somewhat dogmatic views as to their treatment. The author emphasises the tendency to forget that the restriction of the patient to white meat does not solve the dietetic problem of Bright's disease, and points out that a restricted quantity of red meat is both less expensive and less harmful to the patient than the large quantities of white meat frequently permitted. Similarly he calls attention to the high percentage of starch present in many gluten preparations extensively used in the treatment of diabetes. The eye changes, so important in renal affections, are very fully discussed and illustrated by Dr. de Schweinitz.

Premature Burial and How It May be Prevented. By W. Tebb, F.R.G.S., and Col. E. P. Vollum, M.D. Second edition by W. R. Hadwen, M.D. (London: Swan Sonnenschein and Co. Pp. 454. Price 6s.)

Although the dangers of premature burial as set forth in this volume are probably greatly exaggerated, few persons will be disposed to differ from the authors in their criticism of the arrangements for death certification and interment which exist in this country. If the recommendations of the Select Committee of the House of Commons, which met so long ago as 1893, were carried into effect, the granting of death certificates in the case of living persons would become almost an impossibility. Although the early signs of death are individually open to fallacy, taken together they are such that the cases in which any doubt should remain after examination by a medical man would be very few, and in these there can be but one rule—to wait for signs of putrefaction. This, in the larger centres of population, involves the provision of mortuaries, at least for the poor, on whose account they are desirable on many other grounds.

Essay on Insanity. By Charles Williams, L.R.C.P., etc. (London: Henry Glaisher. 1905. Pp. 22. 1s.)

The objects of this essay are in the highest degree commendable. They are to show that insanity is in its very earliest stage much more curable than in its later stages; that attention on the part of the friends and the medical practitioner is requisite for detection of the earliest appearance, and that the most usual symptoms are sleeplessness, loss of memory, headache, suspiciousness, loquacity, absence of judgment, loss of control, changed manner, delusions and hallucinations. Of course, all these are never present at first, and some of them would indicate developed insanity. The real point to look for is that discord between the man and his surroundings, without which insanity never exists. Dr. Williams does not think spiritualists are necessarily insane, and we are disposed to agree with him; but assuredly the spiritualist has tilled the field where insanity is not unlikely to appear as a future crop, and we doubt whether the psychological critic would pronounce a *confirmed* spiritualist *perfectly* sane, although he might not be a certifiable lunatic. We are sure the general practitioner will do well to read this little pamphlet.

A Manual of Toxicology. By A. H. Brundage, A.M., M.D., Phar. D. (London: Baillière, Tindall and Cox. 1904. Pp. 401. Fourth edition. Price 8s. 6d. net.)

That four editions of this manual should have been called for in less than three years is a fact which of itself speaks well for its practical utility. Cases of poisoning, while unfortunately of too frequent occurrence, do not form a large part of the daily work of the individual practitioner and to many the possession of a handy book containing a succinct account of the leading facts in toxicology is a matter of much convenience. Here one may obtain within a moderate compass guidance not only as to symptoms and treatment, tests and post-mortem appearances, but also information as to the unusual effects of drugs, maximum doses of new remedies, incompatibles and much tabular matter. The book is alphabetically arranged and well indexed, while the illustrations, especially the coloured plates, should prove of considerable value.

The Advertisers' A B.C., 1905. (London: Messrs. T. B. Browne, Ltd. Price 10s. 6d.)

The edition for 1905 of this book contains, as usual, a mass of information compiled more especially in the interests of advertisers, journalists, and business men. Such information includes the advertising charges and other particulars, with more or less fulness, of the press of the civilised world. The book is well printed on art paper and contains some excellent examples of present-day illustrations.

### BOOKS RECEIVED.

J. Bale, Son and Danielsson, Limited.

"The Surgical Treatment of Facial Neuralgia." By J. Hutchinson, Jun., F.R.C.S.

Simpkin, Marshall, Hamilton, Kent and Co., Limited.

"The Plenum or Propulsion System of Heating and Ventilating." By Harold Griffiths.

Philip Wellby.

"The Annals of Psychical Science." No. 1. January 1905.

Baillière, Tindall and Cox.

